# Robust Adaptive Beamforming Algorithm Based on Improved Generalized Linear Combination

**DOI:** 10.3390/s26113385

**Published:** 2026-05-27

**Authors:** Zhiqi Gao, Ruyu Zuo, Pingping Huang, Wei Xu, Weixian Tan, Zhixia Wu

**Affiliations:** 1 College of Information Engineering, Inner Mongolia University of Technology, Hohhot 010080, China; gzqngd@imut.edu.cn (Z.G.); hpp@imut.edu.cn (P.H.); xuwei1983@imut.edu.cn (W.X.); wxtan@imut.edu.cn (W.T.); zxwu@imut.edu.cn (Z.W.); 2 Inner Mongolia Key Laboratory of Radar Technology and Application, Hohhot 010051, China

**Keywords:** robust beamforming, generalized linear combination, diagonal loading, singular spectrum analysis, steering vector estimation

## Abstract

Conventional adaptive beamforming algorithms often suffer from significant performance degradation when steering vector mismatches and covariance matrix estimation errors occur. To address this problem, this paper proposes an adaptive robust beamforming algorithm based on an improved generalized linear combination (GLC) framework. The proposed method first applies singular spectrum analysis to the received signals to suppress noise components. A diagonal loading coefficient function related to the received signal snapshots is then constructed, and a generalized diagonally loaded covariance matrix is formed using the denoised data. Finally, by exploiting spatial integration and subspace projection within a predefined angular uncertainty set, the actual direction of arrival of the desired signal is accurately estimated, and the steering vector is corrected accordingly. Simulation results demonstrate that, compared with traditional SMI, LSMI, GLC and improved GLC algorithms, the proposed method achieves a 3–5 dB higher output signal-to-interference-plus-noise ratio (SINR) across the entire input signal-to-noise ratio (SNR) range under steering vector mismatch, and reaches an output SINR close to the optimal level with only 100 snapshots, exhibiting excellent robustness against steering vector mismatch and limited snapshot conditions.

## 1. Introduction

Adaptive beamforming, a core technique in array signal processing, adaptively adjusts the weighting factors of array sensors to effectively enhance the desired signal and suppress interference and noise in the spatial domain. It has been widely adopted in radar, sonar, wireless communications, and medical imaging systems, significantly enhancing their signal processing and anti-interference performance. However, adaptive beamforming typically experiences severe performance degradation in engineering deployments due to model mismatch. Existing algorithms mostly rely on precise prior information regarding propagation models, array manifolds, and signal statistical properties, while imposing idealized constraints on interference environments and operating conditions. Once the actual operating conditions deviate from these prior assumptions, their performance degrades drastically.

Specifically, deviations invariably exist between the actual steering vector of the desired signal and the presumed one, arising from direction-of-arrival estimation errors, amplitude-phase and position errors of array elements, near-far field effects, and coherent or incoherent local scattering effects. Such deviations cause the beamformer to misclassify the desired signal as interference and form a null in its propagation direction, a phenomenon referred to as self-nulling, which leads to a drastic decline in the output signal-to-interference-plus-noise ratio (SINR) [1]. Furthermore, constrained by real-time signal processing requirements, the number of available snapshots for adaptive beamforming is far lower than the theoretical requirement, which degrades the estimation accuracy of the sample covariance matrix (SCM) and thus causes the calculated weight vector to deviate from the optimal solution. In cases where the desired signal component is contained in the sample data, the performance degradation and convergence rate reduction of adaptive beamforming become even more pronounced [2]. Increasing the number of snapshots to alleviate estimation errors leads to a significant increase in computational load, hardware costs, and system power consumption, while compromising real-time responsiveness [3]. This issue is particularly severe in large-scale multi-element arrays, where the complexity of high-dimensional matrix operations grows exponentially. Consequently, there exists an urgent necessity for the development of fast, robust, and cost-effective beamforming algorithms tailored for practical engineering applications.

Extensive research efforts have been devoted to reducing the computational complexity of adaptive beamforming algorithms. A fast linearly constrained robust Capon beamforming algorithm was proposed, based on an iterative gradient method. By employing rank-1 updates and the gradient descent method, the algorithm reduces the time complexity from the conventional O(*M*^3^) to O(*M*^2^), which notably enhances real-time processing performance in single-snapshot update scenarios. This approach drastically cuts down the computational resource requirements without incurring significant performance loss, thereby offering a feasible solution for engineering deployments [4]. Moreover, a variety of robust beamforming algorithms aimed at achieving low sidelobes have been systematically explored, with methods proposed based on quadratic constraints, subspace projection, objective function optimization, and virtual interference sources. These approaches are capable of effectively mitigating beam sidelobe levels and enhancing algorithm robustness in non-ideal scenarios [5], thereby rendering them of significant value for systems functioning in environments with strong interference (e.g., radar and electronic countermeasure systems).

Diagonal loading (DL) is one of the most prevalent robust beamforming techniques. Its core principle is to introduce a loading term into the SCM to improve numerical stability and alleviate performance degradation induced by various errors [6]. An adaptive diagonal loading algorithm, grounded in the eigenstructure of the covariance matrix, has been proposed [7]. This algorithm automatically computes the loading coefficient by leveraging eigenvalue distribution, eliminating the necessity for manual parameter adjustment. This approach effectively overcomes the limitation inherent in traditional fixed loading factors, which are unable to adapt to time-varying scenarios. A method for adaptively selecting the optimal diagonal loading factor was proposed, fusing signal Doppler information, which optimizes the anti-interference performance for acoustic vector array scenarios and further extends the application scope of DL algorithms [8]. This approach optimizes anti-interference performance in acoustic vector array scenarios, thereby broadening the application scope of DL algorithms. The general linear combination (GLC) algorithm, grounded in the minimum mean square error (MMSE) criterion, has emerged as a pivotal research focus for robust beamforming. It has been verified that the GLC algorithm dynamically adjusts the loading factor using the sample covariance matrix (SCM), significantly improving its robustness under limited snapshot conditions [9]. An enhanced GLC algorithm was further developed by introducing a transformation function to address the issues of array element perturbations and steering vector mismatches [10]. Although this improved algorithm achieves a higher output SINR over a wide range of signal-to-noise ratio (SNR) conditions, it still neglects the joint influence of random noise and coherent noise components.

Steering vector estimation provides an effective solution to the model mismatch problem. Researchers have focused on accurately estimating or calibrating the true steering vector to compensate for model deviations induced by angle errors and array perturbations, while ensuring the engineering practicability of the proposed methods. An algorithm based on matrix reconstruction and steering vector search was proposed [11]. By adopting generalized Capon power spectrum analysis and a gradient-guided search strategy, this algorithm facilitates high-precision steering vector estimation, effectively compensating for model mismatches arising from the superposition of multiple error sources. Integrate the sequential quadratic programming into the iterative estimation of the steering vector based on covariance matrix processing, enhancing algorithm robustness in complex mismatch scenarios [12].

Covariance matrix reconstruction stands as another pivotal research avenue for robust adaptive beamforming. By eliminating the desired signal component from the SCM to construct an accurate interference-plus-noise covariance matrix (INCM), the self-nulling phenomenon triggered by desired signal leakage can be effectively circumvented. A covariance matrix reconstruction method rooted in the iterative adaptive algorithm (IAA) was introduced [13]. Through iterative optimization, this approach bolsters the estimation precision of the reconstructed matrix and showcases superior interference suppression capabilities under low-snapshot conditions. The covariance matrix reconstruction was amalgamated with steering vector optimization, by collaboratively designing matrix structure modification and steering vector calibration, this method further augments algorithm robustness in scenarios with multiple errors [14].

In the field of array signal processing and adaptive beamforming, robust algorithms such as Capon beamforming, diagonal loading, adaptive selection, and generalized linear combination (GLC) frameworks aim to optimize receive beam patterns, suppress interference, and enhance output SINR under steering vector mismatch and limited snapshots. This technical logic parallels laser beam shaping, where liquid crystal (LC) phase modulators and deformable mirrors (DM) are dominant solutions: LC modulators enable high-resolution programmable phase control for structured light, while DMs offer high speed and power tolerance for real-time adaptive optics, with their selection guided by application requirements [15]. For semiconductor lasers, one-dimensional beamforming systems correct asymmetric divergence and improve brightness via aspheric lenses, cylindrical optics, or phase modulation [16]. In microelectronics laser micromachining, beamforming transforms Gaussian beams to flat-top or array profiles, ensuring uniform energy distribution, reducing heat-affected zones, and enabling micron/nanoscale processing precision [17]. This paper’s array beamforming methods target receive-end signal optimization, differing fundamentally from transmit-end laser beamforming that modulates optical phase/intensity. Yet both domains share core foundations in wavefront control, spatial filtering, and adaptive optimization—diagonal loading and covariance reconstruction in beamforming inform DM wavefront correction, while phase optimization principles guide LC modulator design.

In summary, researchers have proposed various adaptive beamforming methods centered on diagonal loading, steering vector estimation, and covariance matrix reconstruction. However, existing research still exhibits notable limitations. Firstly, some DL algorithms lack adaptability to scenarios with multiple concurrent errors. Secondly, the accuracy of steering vector estimation needs further improvement under low SNR and strong-interference conditions. Thirdly, most covariance matrix reconstruction algorithms heavily rely on angular prior information and are highly sensitive to parameter variations. To address these aforementioned limitations, this paper proposes a robust adaptive beamforming algorithm based on an improved GLC framework. By synergistically integrating denoising preprocessing, dynamic loading coefficient design, and high-precision steering vector estimation, the proposed algorithm achieves enhanced robustness under complex non-ideal operating conditions, thus providing more reliable theoretical and technical support for practical engineering deployments.

## 2. Signal Model and Basic Principles

### 2.1. Signal Model

**Figure 1:**
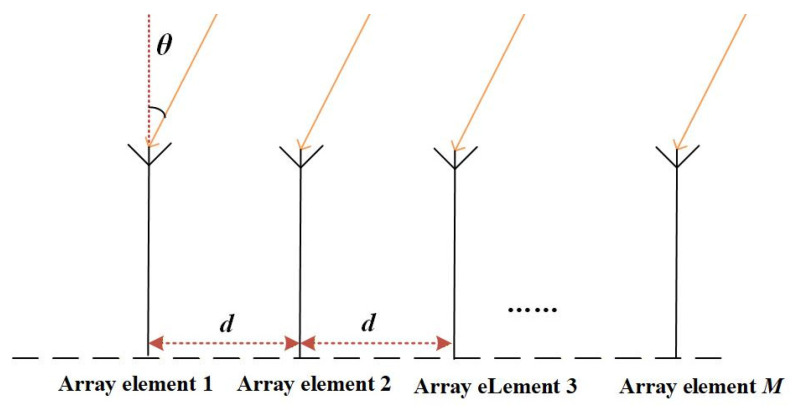
Uniform linear array signal reception model.

The signal receiving model of a uniform linear array (ULA) is shown in Figure 1.

For a uniform linear array with *M* elements, the array receives a narrowband desired signal from direction *θ*_0_ and interference signals from directions *θ*_i_ (i = 1, 2, …, *P*) at time *k*, where *P* + 1 < *M*. The array’s received data ***x***(*k*), named snapshot, is expressed as

(1) $$x\left(k\right)=x_{0}\left(k\right)+x_{i}\left(k\right)+n\left(k\right)$$

where $x_{0}\left(k\right)=s_{0}\left(k\right)a\left(\theta_{0}\right)\in C^{M\times 1}$ denotes the desired signal, $x_{i}\left(k\right)=\sum_{i=1}^{P}s_{i}\left(k\right)a\left(\theta_{i}\right)\in C^{M\times 1}$ is the interference signal; *s*_0_(*k*) and *s_i_*(*k*) represent the waveform envelopes of the desired signal and the interference signal, respectively; *n*(*k*) is additive white Gaussian noise (AWGN) with a mean of 0 and a variance of *σ_n_*^2^. Furthermore, *k* = 1, 2, …, *K*, where *K* is defined as the number of snapshots, which refers to the total number of independent synchronous sampling times of the array received signal in array signal processing. Each snapshot corresponds to an *M*-dimensional column vector ***x***(*k*) collected by all array elements at the same time instant *k*, and *K* snapshots form the complete array received data matrix ***x*** = [***x***(1), ***x***(2), …, ***x***(*K*)]∈ C*^M^*^×*K*^. The snapshot number is a core parameter for estimating the array covariance matrix and implementing adaptive beamforming algorithms [18]. Where, ***a***(*θ*) denotes the steering vector for signals incident on the ULA from the direction *θ*, which is expressed as

(2) $$a\left(\theta \right)={\left[{1,e}^{-j\frac{2\pi d}{\lambda }\sin\theta },\ldots {,e}^{-j\frac{2\pi d}{\lambda }\left(M-1\right)\sin\theta }\right]}^{T}$$

where *λ* is the signal wavelength, *d* is the spacing between adjacent array elements and (·)**^T^** denotes the transpose operation.

The core of beamforming lies in performing linear weighting on the received data by designing the weight vector $w={\left[w_{1},w_{2},\ldots ,w_{M}\right]}^{T}$, and the output signal expressed as

(3) $$y\left(k\right)=w^{H}x\left(k\right)$$

The performance of beamforming algorithms is typically evaluated by the output signal-to-interference-plus-noise ratio (SINR), which is defined as the ratio of the desired signal power to the total interference-plus-noise power at the beamformer output, given by

(4) $$\mathrm{SINR}=\frac{\sigma_{s}^{2}{\left|w^{H}a\left(\theta_{0}\right)\right|}^{2}}{w^{H}R_{i+n}w}$$

where $\sigma_{s}^{2}=E\left\{{\left|s\left(k\right)\right|}^{2}\right\}$ denotes the power of the desired signal; ***w*** is the weighted vector of the beamformer; $R_{i+n}\in C^{M\times M}$ represents the joint covariance matrix of the interference and noise signals, which is defined as $R_{i+n}=E\left\{\left(x_{i}\left(k\right)+n\left(k\right)\right){\left(x_{i}\left(k\right)+n\left(k\right)\right)}^{H}\right\}$; ***a***(*θ*_0_) is the steering vector of the desired signal.

Under the maximum output SINR criterion, adaptive beamforming is equivalent to the following optimization problem [19].

(5) $$\underset{w}{\min}\;w^{H}R_{i+n}w,\;\mathrm{subject}\;\mathrm{to}\;w^{H}a\left(\theta_{0}\right)=1$$

Using the Lagrange multiplier method to solve yields the optimal weight vector under ideal conditions as [20]

(6) $$w_{\mathrm{opt}}=\frac{R_{i+n}^{-1}a(\theta_{0})}{a^{H}(\theta_{0})R_{i+n}^{-1}a(\theta_{0})}$$

In practical applications, the ideal covariance matrix ***R****_i+n_* is difficult to obtain directly, so the sample covariance matrix ${\hat{R}}_{x}$ estimated from finite snapshot data is generally used as a replacement, given by ${\hat{R}}_{x}=\frac{1}{K}\sum_{k=1}^{K}x\left(k\right)x^{H}\left(k\right)$, where *K* denotes the number of snapshots. Solving for the weight vector w under the maximum SINR criterion yields the weight vector for practical applications as

(7) $$w=\frac{{\hat{R}}_{x}^{-1}a(\theta_{0})}{a^{H}(\theta_{0}){\hat{R}}_{x}^{-1}a(\theta_{0})}$$

### 2.2. Basic Principles of Generalized Linear Combination Algorithm

The general linear combination (GLC) algorithm is an adaptive diagonal loading algorithm based on the minimum mean square error (MMSE) criterion. Essentially, it is an enhanced version of the traditional diagonal loading sample matrix inversion (LSMI) algorithm. The conventional LSMI algorithm, which is based on an identical diagonal loading strategy, has its array weight vector specified as

(8) $$w_{\mathrm{LSMI}}=\frac{{\left({\hat{R}}_{x}+\lambda I\right)}^{-1}a(\theta_{0})}{a^{H}(\theta_{0}){\left({\hat{R}}_{x}+\lambda I\right)}^{-1}a(\theta_{0})}$$

where *λ* is the fixed diagonal loading coefficient and ***I*** denotes the *M* × *M*-dimensional identity matrix.

The conventional LSMI algorithm can reduce the spread of small eigenvalues of the sample covariance matrix and enhance the robustness of beamforming [9]. However, the diagonal loading coefficient of traditional diagonal loading algorithms is fixed and challenging to select. Compared to the LSMI algorithm, the core advantage of the GLC algorithm lies in its ability to adaptively determine the diagonal loading coefficient based on the statistical characteristics of the covariance matrix, which avoids the issue of fixed and difficult-to-optimize selection of the loading factor in the LSMI algorithm.

The GLC algorithm achieves adaptive adjustment of the loading coefficient by constructing a linear combination of the sample covariance matrix and the identity matrix [21]. The estimated form of the covariance matrix is given by

(9) $$\tilde{R}={\hat{R}}_{x}+\frac{\alpha }{\beta }I$$

The parameters *α* and *β* are the combination coefficients to be optimized, which can be solved via the MMSE criterion, given by

(10) $$\underset{\alpha ,\beta }{\min}\left\{\mathrm{MMSE}\left({\hat{R}}_{x}\right)\right\}=\underset{\alpha ,\beta }{\min}E\left\{{\left\|\beta {\hat{R}}_{x}+\alpha I-Rx\right\|}^{2}\right\}$$

where $Rx=E\left\{x\left(k\right)x^{H}\left(k\right)\right\}$ denotes the true covariance matrix of the received signal. Throughout the paper, for a vector ***a***, ‖***a***‖ denotes its L_2_ norm; for a matrix ***R***, ‖***R***‖ denotes its spectral norm, i.e., the maximum singular value of ***R***.

For a ULA with *M* array elements, three intermediate parameters *p*, *q*, and *l* are defined as

(11) $$p=\mathrm{tr}({\hat{R}}_{x})/M$$

(12) $$q=E\left\{\left\|{\tilde{R}}_{x}-Rx\right\|\right\}=\frac{1}{N^{2}}\sum_{k=1}^{N}{\left\|x\left(k\right)\right\|}^{4}-\frac{1}{N}{\left\|{\hat{R}}_{x}\right\|}^{2}$$

where ‖·‖^4^ denotes the fourth power of the Euclidean 2-norm for vectors.

(13) $$l={\left\|pI-{\hat{R}}_{x}\right\|}^{2}$$

Solving for the parameters *α* and *β* yields [22]

(14) β=1−q/l

(15) $$\alpha =p\left(1-\beta \right)=qp/l$$

Let ***a***(*θ*_0_) be the steering vector corresponding to the desired signal; then, the weight vector of the GLC algorithm is given by

(16) wGLC=R^+αβI−1a(θ0)aH(θ0)R^+αβI−1a(θ0)

The corresponding output SINR of the beamformer is given by

(17) $${\mathrm{SINR}}_{\mathrm{GLC}}=\frac{\sigma_{0}^{2}{\left|w_{\mathrm{GLC}}^{H}a\left(\theta_{0}\right)\right|}^{2}}{w_{\mathrm{GLC}}^{H}R_{i+n}w_{\mathrm{GLC}}}$$

## 3. Design of Adaptive Beamforming Algorithm Based on GLC

### 3.1. Signal Denoising Preprocessing

In the parameter calculation process of the GLC algorithm, the intermediate parameters *p*, *q* and *l* defined by Formulas (11)–(13) all depend on the estimation accuracy of the sample covariance matrix. Random noise mixed in the actual received signal degrades the statistical characteristics of the covariance matrix, resulting in calculation deviations of the diagonal loading coefficients and ultimately degrading the output performance of the beamformer. To address this issue, this paper adopts the singular spectrum analysis (SSA) method [23] to achieve effective separation of the signal and noise.

Let the noise-containing signal received $x_{m}={\left[x_{m}(0),x_{m}(1),\ldots ,x_{m}(N-1)\right]}^{T}$ by the *m*-th element be (*m* = 1, 2, …, *M*). The sliding window width is set to *L*, which satisfies the constraint condition *L* + *K* = *N* + 1, where *K* denotes the number of constructed vectors, and *L* ≥ *K*. Based on this sliding window, reconstruct the noise-containing signal ***x****_m_* to construct an *L* × *K*-dimensional Hankel matrix ***X***^(*m*)^, whose matrix structure is given by [24]

(18) $$X^{\left(m\right)}=\left[\begin{array}{cccc} x_{m}(0) & x_{m}(1) & \ldots & x_{m}(K-1)\\ x_{m}(1) & x_{m}(2) & \ldots & x_{m}(K)\\ \vdots & \vdots & \ddots & \vdots \\ x_{m}(L-1) & x_{m}(L) & \ldots & x_{m}(N-1) \end{array}\right]$$

Perform singular value decomposition on the Hankel matrix ***X***^(*m*)^. Based on the cumulative proportion of singular value energy, retain the main components of the signal that account for 92% of the total energy, and eliminate the noise components [25]. The Hankel matrix ***X***^(*m*)^ can be decomposed into the sum of vectors spanning the signal subspace ***X****_ti_*^(*m*)^ and vectors spanning the noise subspace ***X****_n_*^(*m*)^, given by ***X***^(*m*)^ = ***X****_ti_*^(*m*)^ + ***X****_n_*^(*m*)^. Performing singular value decomposition (SVD) on ***X***^(*m*)^ yields

(19) $$X^{\left(m\right)}=\left[\begin{array}{cc} G_{1} & G_{2} \end{array}\right]\left(\begin{array}{cc} \Sigma_{1} & 0\\ 0 & \Sigma_{2} \end{array}\right)\left[\begin{array}{c} P_{1}^{T}\\ P_{2}^{T} \end{array}\right]$$

where $G_{1}\in C^{L\times D_{0}}$ is the left singular vector matrix spanning the signal subspace, $\Sigma_{1}\in C^{D_{0}\times D_{0}}$ is the diagonal matrix composed of the singular values of the vectors spanning the signal subspace, and $P_{1}\in C^{K\times D_{0}}$ is the right singular vector matrix spanning the signal subspace. ***G***_2_, ***Σ***_2_, ***P***_2_ correspond to the singular value decomposition results of the vectors spanning the noise subspace. Based on the above decomposition, vectors spanning the signal subspace matrix can be expressed as

(20) $$X_{ti}^{\left(m\right)}=G_{1}\times \Sigma_{1}\times P_{1}^{H}$$

The diagonal averaging method is applied to reconstruct ***X****_ti_*^(*m*)^ into a denoised sequence of the same length as the original signal. Let *y_ij_*^(*m*)^ denote the element at the *i*-th row and *j*-th column of ***X****_ti_*^(*m*)^ (with 1 ≤ *i* ≤ *L*, 1 ≤ *j* ≤ *K*). Then, each element of the reconstructed denoised sequence $y_{rc}^{\left(m\right)}=\left[y_{rc1}^{\left(m\right)},\cdots ,y_{rck}^{\left(m\right)},\cdots y_{rcN}^{\left(m\right)}\right]$ is calculated by the following piecewise function.

(21) $$y_{rck}^{\left(m\right)}=\left\{\begin{array}{ll} \frac{1}{k}\sum_{m=1}^{k}y_{m,k-m+1}^{\left(m\right)} & \left(1\leq k<K\right)\\ \frac{1}{K}\sum_{m=1}^{K}y_{m,k-m+1}^{\left(m\right)} & \left(K\leq k\leq L\right)\\ \frac{1}{N-k+1}\sum_{m=k-L+1}^{K}y_{m,k-m+1}^{\left(m\right)} & \left(L<k\leq N\right) \end{array}\right.$$

Performing the above processing on all array elements yields the global denoised received signal and its corresponding covariance matrix as

(22) $$y_{rc}(n)=[y_{rc}^{\left(0\right)}(n),y_{rc}^{\left(1\right)}(n),\cdots ,y_{rc}^{\left(M-1\right)}(n){]}^{T}$$

(23) $$R_{yrc}=\frac{1}{N}\sum_{n=1}^{N}y_{rc}(n)y_{rc}^{H}\left(n\right)$$

### 3.2. Improvement of Loading Coefficient in GLC Algorithm

The diagonal loading coefficient expression of the GLC algorithm is [24]

(24) $$DL=\frac{\alpha }{\beta }=\frac{p}{l/q-1}$$

It should be emphasized that the parameters *p*, *q*, and *l* in the proposed diagonal loading scheme are all closely related to the number of snapshots *N*, even though this dependence is not explicitly shown in a simple algebraic form. The sample covariance matrix ***R****_yrc_* is estimated from *N* received vectors, and its estimation error decreases significantly as *N* increases. The parameter *p* represents the average power of array outputs, whose statistical accuracy improves with increasing *N*. The parameter q reflects fourth-order statistical properties of the received data and the discrepancy between sample and true statistics, which is also determined by the finite sample size. Similarly, *l* characterizes the overall deviation of the sample covariance matrix from a scaled identity matrix, which decreases monotonically as the reliability of ***R****_yrc_* improves with more snapshots.

**Figure 2:**
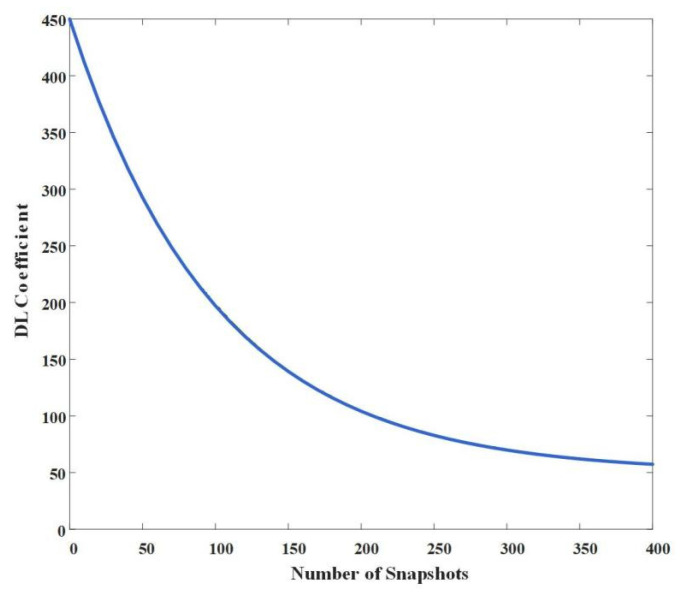
Relationship between the number of snapshots and the diagonal loading coefficient.

In the GLC algorithm, the variation of the DL coefficient with the number of snapshots is depicted in Figure 2 [9]. If the DL coefficient is properly selected, the performance of the GLC algorithm can gradually approach that of the sample matrix inversion (SMI) algorithm [26]. However, the performance of the GLC algorithm is sensitive to the selection of the DL coefficient, and it degrades in the presence of array errors or steering vector mismatch [27]. Therefore, it is necessary to improve the selection method of the DL coefficient for the GLC algorithm to enhance the robustness of the algorithm.

To address the severe performance degradation in small-snapshot scenarios, a conversion function *C*(*N*) is introduced, which shows an exponential-like transition with *N*. This design is based on the classical fact that the sample covariance matrix is heavily biased when *N* is small, and stronger regularization is required to suppress estimation errors. As *N* increases, the estimation accuracy improves rapidly, and the intensity of diagonal loading can be reduced accordingly.

Furthermore, a snapshot conversion threshold *N*_fix_ is introduced it is chosen as a critical snapshot number that distinguishes small-snapshot regimes from asymptotic large-snapshot regimes in adaptive beamforming [28]. According to classical sampling theory in array processing, the sample covariance matrix becomes statistically reliable only when *N* is sufficiently larger than the number of array elements *M* [29]. In practice, *N*_fix_ is typically selected in the range from 2*M* to 4*M* according to empirical and theoretical studies. In this work, *N*_fix_ = 32 is adopted, which is a moderate value within this range and is verified to provide stable transition performance for arrays with *M* = 16 elements.

The rate adjustment coefficient *t* ∈ [0, 1] controls the convergence speed of the adaptive factor *C*(*N*) as *N* increases. A smaller *t* leads to slower variation, while a larger *t* leads to faster convergence. Based on extensive simulations and existing adaptive regularization designs [8], *t* = 0.2 is chosen to achieve a reasonable balance between adaptation speed and algorithm stability. This value ensures that the regularization strength decreases gently as the snapshot number grows, avoiding excessive loading in large-snapshot situations while maintaining sufficient robustness when *N* is small. Both parameters are determined from a systematic simulation study over typical working conditions, including different SNR levels, interference scenarios, and array sizes, which confirms their effectiveness and generality. The conversion function formula is

(25) $$C(N)=\frac{N^{t}}{1+\exp\left(-28(N-N_{\mathrm{fix}})\right)}+1$$

Based on the conversion function, a coefficient correction strategy is formulated. When *N* ≤ *N*_fix_, the original method is adopted to calculate the intermediate parameter *l*; when *N* > *N*_fix_, the intermediate parameter *l* is adaptively modified as

(26) $$\hat{l\left(N\right)}=\frac{l}{C(N)}=\frac{{\left\|pI-R_{yrc}\right\|}^{2}}{C(N)}$$

Finally, the dynamic adaptive diagonal loading coefficient is obtained as

(27) $$\hat{\mathrm{DL}\left(N\right)}=\left\{\begin{array}{ll} \frac{p}{\hat{l}/q-1} & \left(N\leq N_{\mathrm{fix}}\right)\\ \frac{p}{\hat{l}/q-1}C(N) & \left(N>N_{\mathrm{fix}}\right) \end{array}\right.$$

### 3.3. Steering Vector Estimation

From Formula (4), it is evident that any mismatch between the array beam pointing and the actual incident direction of the desired signal will result in a beam main lobe offset or insufficient null depth, thereby severely degrading the output SINR [30]. To enhance the real-time performance and accuracy of steering vector estimation, a search method is employed instead of the traditional correction approach. The optimal steering vector is identified by conducting a traversal search within a reasonable angular range.

Based on the prior information of direction of arrival (DOA) estimation, the angular uncertainty set of the signal of interest (SOI) is defined as $\Theta =\left[\theta_{0}-\Delta \theta ,\theta_{0}+\Delta \theta \right]$, where *θ*_0_ is the initial estimated angle of the SOI and ∆*θ* is the angular search range. This range not only covers the possible angular estimation errors but also avoids the increase in complexity caused by excessive search [31].

A uniform sampling search strategy is adopted within the uncertainty set Θ, with the sampling step set as *δ*, generating *Q* = [2∆*θ*/*δ*] + 1 candidate angles: *θ_z_* = *θ*_0_ − ∆*θ* + *z*∙*δ* (*z* = 0, 1, …, *Q* − 1).

For each candidate angle *θ_z_*, the corresponding candidate steering vector is generated based on the array structure as

(28) $$a(\theta_{z})={\left[1,e^{j\frac{2\pi d}{\lambda }\sin\theta_{z}},\ldots ,e^{j\frac{2\pi d}{\lambda }\left(M-1\right)\sin\theta_{z}}\right]}^{T}$$

The angular search range ∆*θ* = 5° is determined based on the practical characteristics of steering vector mismatch and direction-of-arrival (DOA) estimation errors. In engineering scenarios such as radar and wireless communications, the nominal incident angle often deviates from the true value due to array calibration errors, atmospheric turbulence, or platform motion, but such deviations are typically within ±5°for narrowband far-field signals [32]. A smaller ∆*θ* may fail to cover the true incident angle, leading to severe mismatch; a larger ∆*θ* will expand the search space unnecessarily, increasing computational redundancy without significant accuracy improvement [33]. Extensive simulations confirm that ∆*θ* = 5° can fully cover typical mismatch scenarios while avoiding excessive computational overhead, which is consistent with the optimal search range selection criterion in robust beamforming.

The search step size *δ* = 0.2° is designed to balance estimation precision and complexity. The step size directly determines the resolution of the angular grid: a smaller *δ* (e.g., 0.1°) improves estimation accuracy but exponentially increases the number of search points *Q* (e.g., *Q* = 101 for *δ* = 0.1° vs. *Q* = 51 for *δ* = 0.2°), leading to higher computational complexity (*O*(*QM*^3^)) [34]. A larger *δ* (e.g., >0.5°) reduces complexity but degrades the resolution of DOA estimation, resulting in residual mismatch. According to the trade-off principle of grid search in array signal processing, *δ* = 0.2° is an optimal choice, it ensures that the estimation error is within 0.1°while keeping the number of search points moderate, and the additional complexity is acceptable since *Q* is a fixed constant that does not increase with the number of array elements *M*.

Using the maximization of the beamforming output SINR as the screening criterion, the objective function is constructed by combining it with the improved diagonal loading coefficient $\hat{DL}$. Firstly, the beamforming weight vector is calculated based on the denoised covariance matrix ***R****_yrc_* and the improved diagonal loading coefficient $\hat{\mathrm{DL}}$ as

(29) $$w_{z}=\frac{a(\theta_{z}){\left(R_{yrc}+\hat{\mathrm{DL}}I\right)}^{-1}}{a^{H}(\theta_{z}){\left(R_{yrc}+\hat{\mathrm{DL}}I\right)}^{-1}a(\theta_{z})}$$

The further constructed objective function is

(30) $$J(z)=\frac{\sigma_{s}^{2}{\left|w_{z}^{H}a(\theta_{z})\right|}^{2}}{w_{z}^{H}R_{yrc}w_{z}}$$

Traversing all candidate angles to find the candidate steering vector that maximizes the objective function *J*(*z*) yields the optimal steering vector $\hat{a}(\theta_{0})=arg\underset{z\in [0,Q-1]}{max}J(z)$.

This search strategy bypasses the intricate convex optimization solution or iterative correction process by traversing through a limited set of candidate angles. This simplifies the implementation process while maintaining estimation accuracy, making it more apt for engineering application scenarios.

In conclusion, this method achieves high-precision estimation and correction of the true steering vector of the desired signal by uniformly sampling within the uncertain angle set Θ and following the output SINR maximization criterion. It completely avoids the complex convex optimization solution and iterative correction process throughout the process, while significantly simplifying the engineering implementation procedure, while ensuring the estimation accuracy. This strategy is based on the covariance matrix ***R****yrc* after SSA noise reduction and the improved dynamic diagonal loading coefficient. It first solves the beamforming weighted vector ***w***_GLC_ through Formula (29). Then, all the candidate orientation vectors ***a***(*θ*_z_) generated by Formula (28) are traversed and filtered, and finally, the optimal orientation vector $\hat{a}(\theta_{0})$ is determined.

Substitute this optimal steering vector into the formula for calculating the signal-to-interference-plus-noise ratio (SINR), and you can obtain the final output SINR of the beamformer as

(31) $$\mathrm{SINR}=\frac{\sigma_{s}^{2}{\left|w_{GLC}^{H}\hat{a}(\theta_{0})\right|}^{2}}{w_{GLC}^{H}R_{\mathrm{yrc}}w_{GLC}}$$

### 3.4. Analysis of Computational Complexity

Table 1 compares the computational complexity of different beamforming algorithms. SMI beamformer serves as the basic scheme, with its main computational load being the inversion of the sample covariance matrix, which has a complexity of *O*(*M*^3^). The LSMI algorithm adds diagonal loading operations on top of SMI, without changing the complexity order of the inversion operation, still remaining at *O*(*M*^3^). The GLC algorithm requires additional calculations of intermediate parameters *p*, *q*, and *l*, but the matrix operation volume remains *O*(*M*^2^), maintaining the overall complexity at *O*(*M*^3^). The improved GLC algorithm introduces singular spectrum analysis for noise reduction, where *n* is the number of snapshots, *L* is the sliding window length of the Hankel matrix, and the complexity of performing SVD decomposition for each array element is *O*(*L*^2^*n*), for a total of *O*(*MnL*^2^) for M array elements, and the overall complexity after matrix inversion is *O*(*M*^3^ + *MnL*^2^). The computational cost of the proposed algorithm mainly lies in the spatial spectrum search *O*(*M*^2^*Q*) (where *Q* is the number of search points in the angular sector) and eigenvalue decomposition, with the overall complexity being *O*(max(*M*^2^*Q*, *M*^3^)).

**Figure 3:**
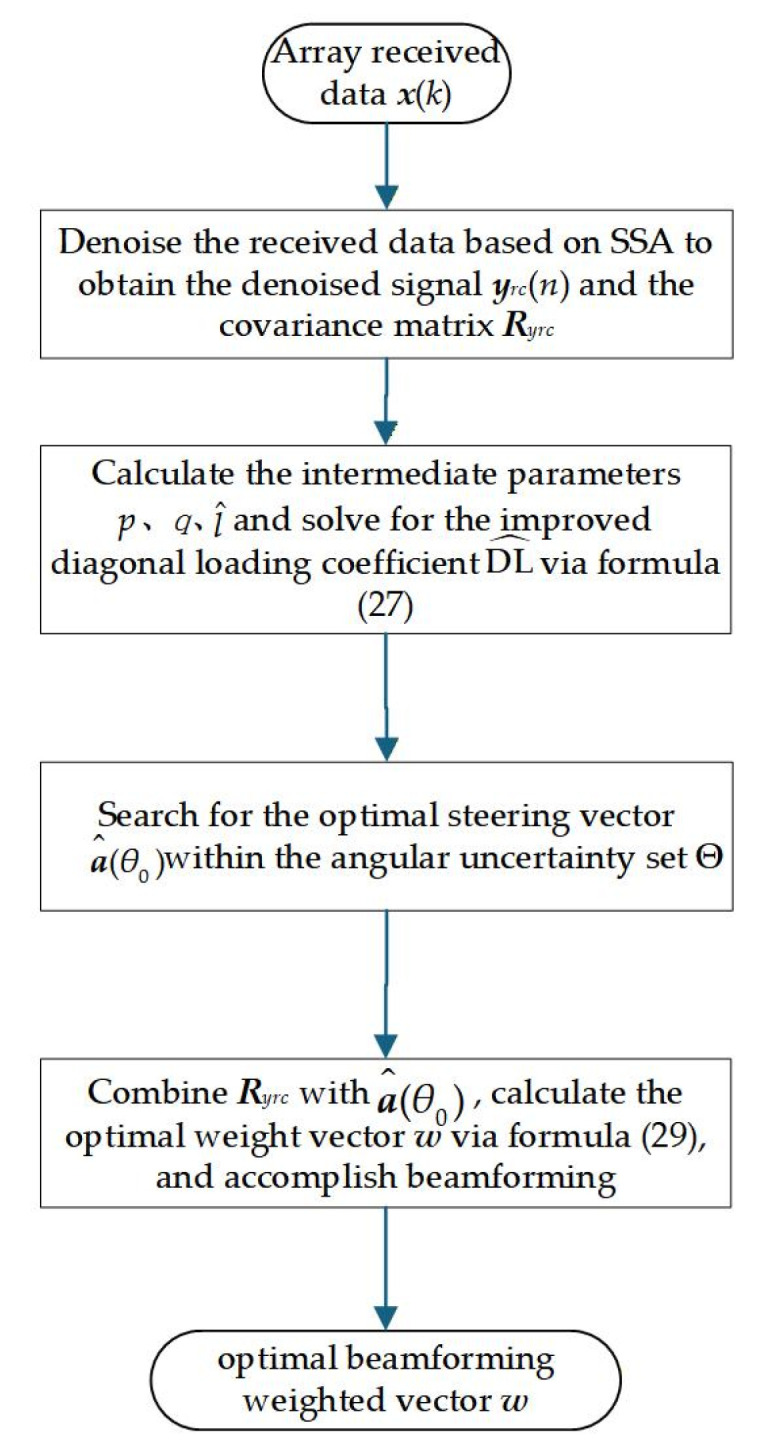
The flowchart of the proposed algorithm.

The overall process of the proposed algorithm is shown in Figure 3.

## 4. Simulation Analysis

### 4.1. Parameter Settings

The simulation constructs the experimental environment based on a standardized array signal processing framework, with the specific parameter configurations as follows: the array is a ULA with 16 elements, the signal carrier wavelength is *λ* = 0.3 m, and the corresponding carrier frequency is *f*_0_ = 1 GHz. The desired signal is set as a far-field narrowband random signal with its nominal incident direction set as *θ*_s_ = 10°; a 2° angular estimation error is introduced to construct a steering vector mismatch scenario. Two independent far-field narrowband random interference signals are considered, which are incident from the directions of *θ_i_*_1_ = −20° and *θ_i_*_2_ = 40°, respectively, with their interference-to-noise ratios (INR) set to 20 dB and 30 dB to simulate a strong interference environment in practical applications. Furthermore, the LSMI algorithm is configured with a load factor of 10 dB, and the actual incident angle range of the desired signal is specified. The rate adaptation coefficient of the transformation function *t* is set to 0.2.

To ensure the fairness and uniformity of the experimental comparisons, all three sets of simulation experiments in this paper adopt the same baseline parameter settings: the interference signal-to-noise ratios (INR) of the two channels are fixed at 20 dB and 30 dB, respectively, and the baseline input signal-to-noise ratio (SNR) is uniformly set at 0 dB. Only in the corresponding experiments are the input SNR range, angle error range, and the number of snapshots changed, respectively.

### 4.2. Impact of Incident Angle Error of Desired Signal on Output SINR

The first experiment examines the robustness of each algorithm against the steering vector of the desired signal. The array received noise is zero-mean space-time complex Gaussian white noise, which is uncorrelated with both the desired signal and interference signals, and the reference SNR is fixed at 0 dB. In the simulation experiments, the number of snapshots is 400, and the input SNR varies in the range of −20 dB to 20 dB, covering the full scenarios of low, medium, and high SNR. The compared algorithms include the SMI algorithm, LSMI algorithm, GLC algorithm, improved GLC algorithm [24], and the proposed algorithm in this paper. Under both scenarios of steering vector matching and mismatch, the robustness and adaptability of the algorithms are quantitatively evaluated by comparing the variation patterns of their output Signal-to-Interference and Noise Ratio (SINR).

The performance divergence among GLC, Improved GLC, and the proposed algorithm arises from their distinct regularization and noise suppression mechanisms. The original GLC relies on a fixed diagonal loading scheme, which cannot adapt to varying SNR conditions, limiting its performance at medium-to-high input SNR. The Improved GLC enhances covariance estimation but still lacks the adaptive steering vector correction of the proposed method, resulting in a persistent performance gap. In contrast, the proposed algorithm combines SSA denoising, dynamic diagonal loading, and steering vector correction to effectively suppress noise distortion and adaptively optimize beamforming weights, enabling it to achieve the highest output SINR across all input SNR levels, with the advantage becoming more pronounced as SNR increases.

**Figure 4:**
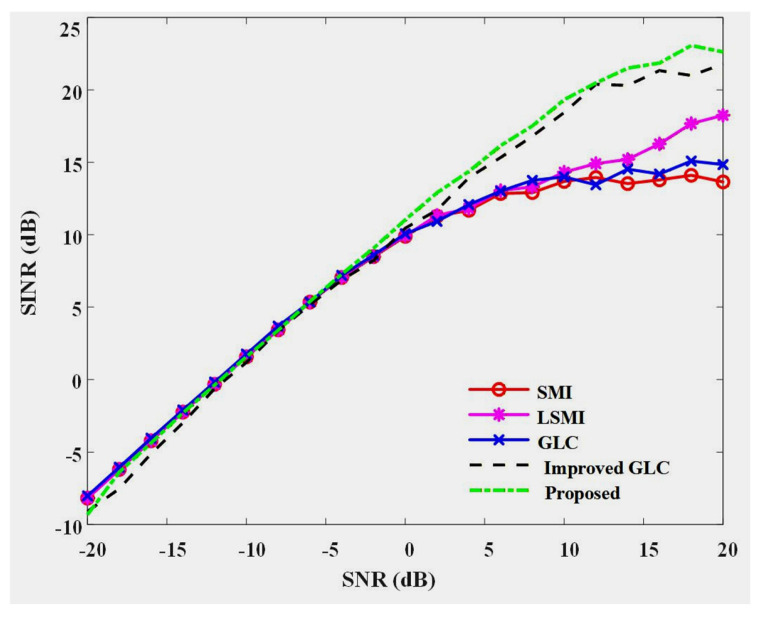
Variation of output SINR with input SNR under steering vector matching.

Figure 4 illustrates the variation of output SINR with input SNR under the steering vector matching scenario. It is evident that as the input SNR gradually increases, the output SINR of all algorithms exhibits a continuous upward trend. Notably, the algorithm proposed in this paper maintains an approximately maximum output SINR across the entire range of input SNR. For the improved GLC and the GLC, at very low SNR from −20 to −15 dB, the sample covariance matrix is severely contaminated by strong noise. The improved GLC introduces SSA-based covariance reconstruction, but the accuracy is reduced due to noise, and hence, there is a performance deviation from the traditional GLC. In the moderate SNR region from 5 to 10 dB, desired signal leakage appears in the sample covariance matrix, causing noticeable performance degradation in the GLC, while the improved GLC can reduce this effect to a certain extent, leading to better performance than that of the GLC algorithm.

**Figure 5:**
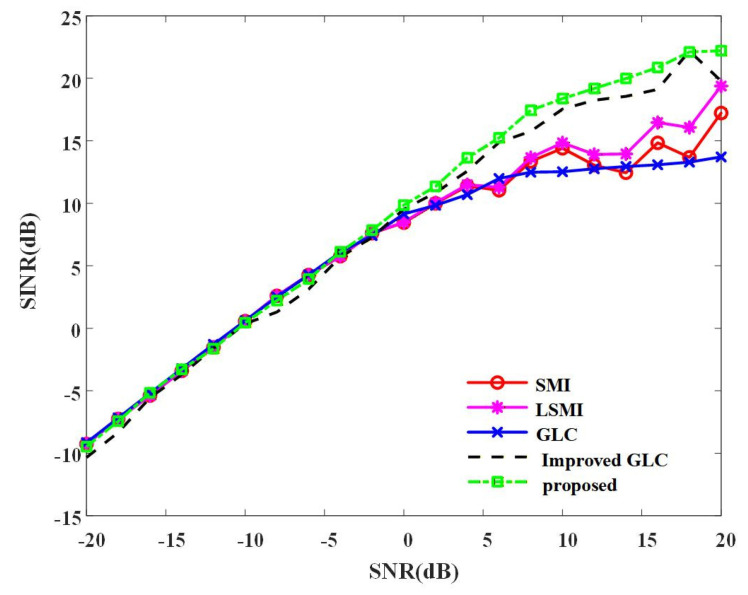
Variation of output SINR with input SNR under steering vector mismatching.

In the scenario of steering vector mismatch, an angular deviation of 2° is set between the actual incident direction and the estimated direction of the desired signal. The variation of output SINR with input SNR is depicted in Figure 5. It can be observed that the performance differentiation among various algorithms becomes more pronounced with increasing input SNR, fully exposing the robustness flaws of traditional algorithms under steering vector mismatch. For the SMI algorithm, once the input SNR surpasses 0 dB, the growth rate of output SINR decelerates sharply, and severe performance deterioration occurs in the high SNR range (SNR > 10 dB), as it cannot effectively mitigate the combined effects of interference and angular errors. The LSMI algorithm also suffers from a diminished output SINR growth rate under medium and high SNR conditions, resulting in limited performance enhancement. The GLC is not as good as that of the above two algorithms, while the improved GLC, despite certain optimizations, still sees its output SINR growth slow down and fluctuate under high SNR due to the lack of effective mismatch suppression. In contrast, the proposed algorithm demonstrates optimal against angle mismatch resistance performance across the entire input SNR range, with its output SINR consistently surpassing all compared algorithms and maintaining a steady upward trend without any noticeable degradation, benefiting from its integrated SSA denoising, dynamic diagonal loading, and steering vector correction design.

### 4.3. Impact of Different Angular Errors on Output SINR

The second experiment examines the robustness of each beamforming algorithm against mismatch resistance, under dynamic changes in the steering vector mismatch degree. The input SNR is set at 0 dB, and the number of snapshots is 400. As the angular error magnitude of the desired signal’s arrival direction varies uniformly within the range of −5° to 5°, we observe the trends in output SINR for each algorithm under different angular errors. This allows for a quantitative evaluation of the algorithms’ robustness and adaptability to angular estimation errors.

**Figure 6:**
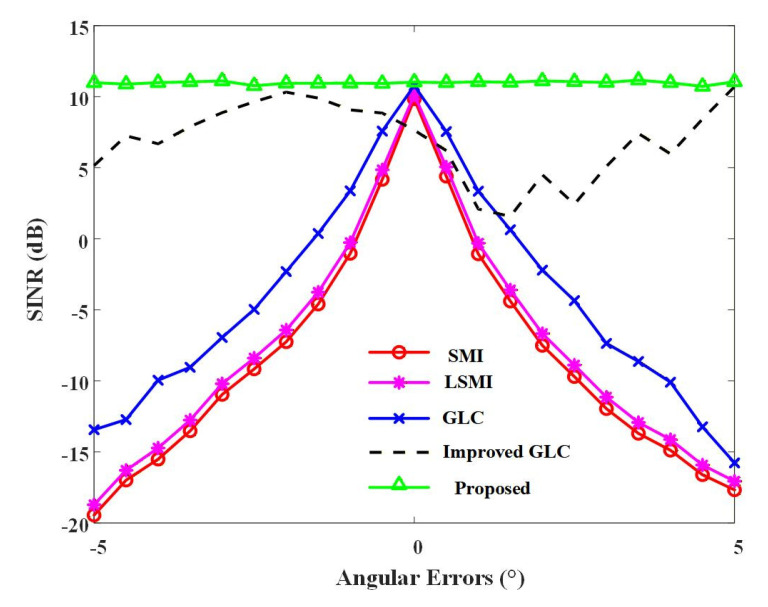
Output SINR under different angular errors.

The output SINR of each algorithm under different angular errors is depicted in Figure 6. It can be seen that the increase in angular error exerts a differentiated impact on the output performance of each algorithm: for algorithms such as SMI, LSMI, and GLC, the output SINR decreases significantly with large angular errors; the improved GLC algorithm exhibits robustness to angular errors but with fluctuations in its output SINR. In comparison, the algorithm proposed in this paper achieves a significant improvement in robustness to angular estimation errors through a multi-dimensional collaborative optimization design. It can maintain stable and high output SINR performance over the wide angular error range of −5° to 5°, and its robustness is significantly superior to that of traditional algorithms and existing improved algorithms.

### 4.4. Impact of Signal Snapshot Number on Output Performance

The third experiment examines the performance of each beamforming algorithm in scenarios with limited sample data volume, particularly focusing on the robustness against large estimation errors in the covariance matrix caused by a low number of snapshots. The input SNR is set to 0 dB, and the number of snapshots is uniformly varied within the range of 20 to 400. This experiment analyzes the output SINR of each algorithm as the number of snapshots varies, both in scenarios with steering vector matching and mismatch, in order to quantitatively assess the algorithms’ dependence on sample data volume.

**Figure 7:**
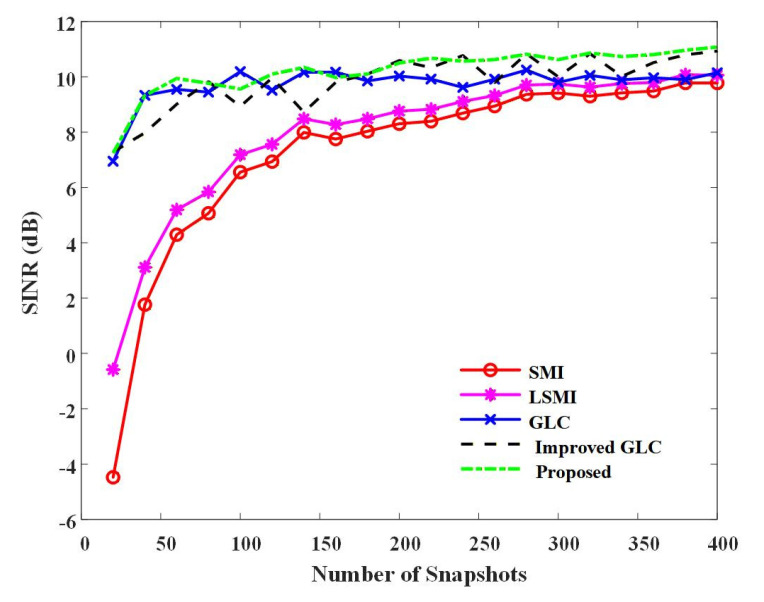
Variation of Output SINR with Number of Snapshots Under Steering Vector Matching.

Figure 7 illustrates the output SINR of each algorithm as the snapshot number varies under steering vector matching. The proposed algorithm achieves a substantial performance advantage over the conventional SMI, LSMI, GLC, and Improved GLC methods, especially in the low-snapshot regime *K* < 100. Notably, when the snapshot count reaches only 100, the output SINR of the proposed algorithm already approaches the asymptotically optimal level, maintaining a steady upward trend and consistently outperforming all benchmark algorithms across the entire snapshot range. It is observed that the output SINR of the proposed algorithm is roughly comparable to that of the original GLC algorithm when *K* < 50. This is because the ultra-limited snapshots lead to large estimation errors in the sample covariance matrix, which limits the performance gain of the proposed algorithm’s dynamic optimization modules, while the fixed strong regularization of GLC compensates for the sample deficiency to a certain extent. As K increases beyond 50, the proposed algorithm’s adaptive mechanism takes effect, pulling away from GLC and other benchmarks. For the compared algorithms, SMI and LSMI suffer from severe performance degradation in the low-snapshot range due to large covariance matrix estimation errors, with their SINR rising slowly and remaining far below the proposed algorithm. The original GLC shows moderate performance in small snapshots but converges slowly, while the Improved GLC outperforms traditional methods but still lags behind the proposed algorithm in both convergence speed and final SINR level.

**Figure 8:**
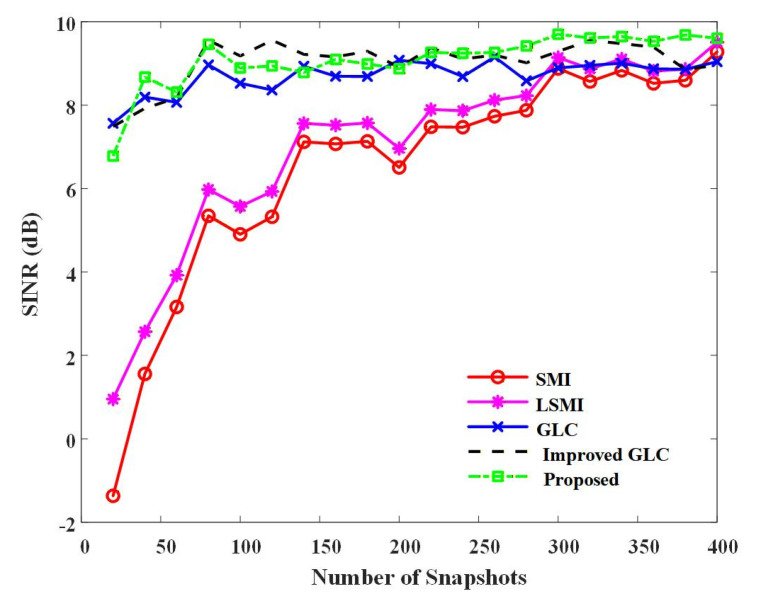
Variation of Output SINR with Number of Snapshots Under Steering Vector Mismatching.

Figure 8 illustrates the output SINR of each algorithm as the snapshot number varies under steering vector mismatch. Evidently, the performance gap between algorithms widens, and the combined effect of a small number of snapshots and mismatch errors exacerbates the performance degradation of traditional algorithms. In contrast, the proposed algorithm maintains excellent adaptability in mismatch scenarios. Even with only 60 snapshots, the output SINR reaches a satisfactory level and continues to steadily increase as the number of snapshots grows. Despite the dual effects of a small number of snapshots and mismatch errors, it effectively mitigates performance degradation.

### 4.5. Discussion

Based on the comparative analysis of simulation results, the proposed robust adaptive beamforming algorithm based on improved generalized GLC demonstrates superior performance in terms of output SINR, robustness against steering vector mismatch, and adaptability under limited snapshot conditions compared with traditional SMI, LSMI, and GLC algorithms. The integrated singular spectrum analysis denoising preprocessing effectively suppresses random noise and reduces the statistical distortion of the sample covariance matrix, while the dynamically optimized diagonal loading coefficient overcomes the poor adaptability of fixed loading factors in conventional GLC methods. The steering vector estimation strategy, combining fine-grid Capon spectrum search and robust Capon beamforming correction, accurately compensates for the mismatch caused by angle estimation errors and array perturbations without complex convex optimization. Moreover, the proposed algorithm maintains the same dominant computational complexity of *O*(*M*^3^) as traditional methods, ensuring good engineering practicability for real-time array systems.

Nevertheless, the method still has some limitations. Firstly, key parameters such as the SSA energy threshold, Hankel matrix constraints, and dynamic loading coefficients are currently determined by empirical simulation, lacking a rigorous adaptive adjustment mechanism for non-stationary noise and time-varying signal environments. Secondly, the fixed angular search grid for steering vector estimation may cause redundant computation in small error scenarios or fail to cover the actual incident direction in large mismatch cases, affecting estimation efficiency and accuracy. Thirdly, the algorithm is currently verified only for narrowband far-field signals based on uniform linear arrays, and its adaptability to broadband signals, near-field targets, and non-uniform arrays remains to be further studied.

## 5. Conclusions

To address the performance degradation of traditional adaptive beamforming algorithms under scenarios such as steering vector mismatch, covariance matrix estimation errors, and a limited number of snapshots, this paper proposes a robust adaptive beamforming algorithm based on improved general linear combination (GLC). The core novelty lies in the organic integration and joint optimization of SSA denoising, snapshot-adaptive diagonal loading, and steering vector search within a unified GLC framework, which distinguishes it from existing methods that only apply these techniques separately.

Simulation experiments with a 16-element uniform linear array verify the superior performance of the proposed algorithm. Compared with traditional SMI, LSMI, GLC and improved GLC algorithms, the proposed method achieves a 3–5 dB higher output SINR across the entire input SNR range of −20 dB to 20 dB under 2° steering vector mismatch, and effectively avoids the performance degradation of traditional algorithms in the high SNR range. In limited snapshot scenarios, the proposed algorithm can reach an output SINR close to the asymptotically optimal level with only 100 snapshots under steering vector matching, and still maintain a satisfactory output SINR with merely 60 snapshots under steering vector mismatch. For angular estimation errors in the range of −5° to 5°, the proposed algorithm stably maintains high output SINR without obvious performance fluctuation, showing significant robustness against large-angle mismatch compared with existing algorithms. In conclusion, the proposed algorithm significantly enhances the beamforming robustness under complex non-ideal scenarios, providing an effective technical solution for engineering applications such as airborne radar and wireless communications.

Future research will focus on developing more adaptive parameter optimization frameworks to enhance the algorithm’s robustness in complex non-stationary environments, and exploring intelligent variable-grid search strategies to reduce computational redundancy while ensuring estimation accuracy. Multi-module joint optimization mechanisms will also be investigated, incorporating covariance matrix reconstruction and low sidelobe beamforming technologies to further improve the algorithm’s anti-interference performance, and extending the framework to broadband and polarization-sensitive array scenarios to expand its application in radar, sonar, and wireless communication systems.

## Figures and Tables

**Table 1:** Computational Complexity.

Beamforming Algorithms	Computational Complexity
SMI	*O*(*M*^3^)
LSMI	*O*(*M*^3^)
GLC	*O*(*M*^3^)
Improved GLC	*O*(*M*^3^ + *MnL*^2^)
Proposed	*O*(max(*M*^2^*Q*, *M*^3^))

## Data Availability

Data is contained within the article.
